# Scaffold-Free Bioprinter Utilizing Layer-By-Layer Printing of Cellular Spheroids

**DOI:** 10.3390/mi10090570

**Published:** 2019-08-29

**Authors:** Wesley LaBarge, Andrés Morales, Daniëlle Pretorius, Asher M. Kahn-Krell, Ramaswamy Kannappan, Jianyi Zhang

**Affiliations:** 1 Department of Biomedical Engineering, School of Medicine, School of Engineering, University of Alabama at Birmingham, Birmingham, AL 35294, USA; 2 Department of Mechanical Engineering, School of Engineering, University of Alabama at Birmingham, Birmingham, AL 35294, USA

**Keywords:** tissue engineering, cell, bioprinting, spheroids

## Abstract

Free from the limitations posed by exogenous scaffolds or extracellular matrix-based materials, scaffold-free engineered tissues have immense clinical potential. Biomaterials may produce adverse responses, interfere with cell–cell interaction, or affect the extracellular matrix integrity of cells. The scaffold-free Kenzan method can generate complex tissues using spheroids on an array of needles but could be inefficient in terms of time, as it moves and places only a single spheroid at a time. We aimed to design and construct a novel scaffold-free bioprinter that can print an entire layer of spheroids at once, effectively reducing the printing time. The bioprinter was designed using computer-aided design software and constructed from machined, 3D printed, and commercially available parts. The printing efficiency and the operating precision were examined using Zirconia and alginate beads, which mimic spheroids. In less than a minute, the printer could efficiently pick and transfer the beads to the printing surface and assemble them onto the 4 × 4 needles. The average overlap coefficient between layers was measured and found to be 0.997. As a proof of concept using human induced pluripotent stem cell-derived spheroids, we confirmed the ability of the bioprinter to place cellular spheroids onto the needles efficiently to print an entire layer of tissue. This novel layer-by-layer, scaffold-free bioprinter is efficient and precise in operation and can be easily scaled to print large tissues.

## 1. Introduction

With the development of cutting-edge strategies and techniques for generating complex, functional tissues, bioprinting, or three-dimensional (3D) printing of biological materials, has become a critical tool in tissue engineering [1,2,3,4,5,6,7]. Bioprinted and fabricated tissues have immense clinical potential including organ and tissue regeneration, drug testing, organ models for surgical practices, and organ replacement. The conventional method of bioprinting is to print cells with or into a scaffold material. Scaffold printing, with either a synthetic or natural polymer, can provide the necessary structure and microenvironment for proper cell growth and survival [8,9,10]. This method can be used to dictate how structures are arranged, where cells are positioned in this scaffold, and what sort of factors are present to optimize the growth and function of the cells. Polymers can also be designed to degrade depending on a specific set of environmental conditions or with the introduction of a particular solution. However, with synthetic polymers, biocompatibility could pose problems if their use is not appropriately addressed in in vivo studies, leading to an increased use of natural polymers and hydrogels for bioprinting [11,12,13,14,15]. These types of materials possess the same ability to provide an adequate structure for the cells and can be designed to mimic the in vivo environment better, decreasing the chances of rejection if used in the body. 

Cells, however, possess the innate ability to produce their scaffold material in the form of extracellular matrix (ECM) as they grow and interact with one another [16,17,18,19]. Determining how to harness this ability and become completely scaffold-free effectively has led to the use of 3D spheroids as building blocks for larger tissues [20]. Spheroids can easily fuse together when in close proximity to or touching another spheroid. The efficacy of spheroids as building blocks for larger tissues has been demonstrated using scaffold materials to provide a structure or a particular shape for the proper fusing of spheroids [21]. However, this method still relies on additional materials for tissue generation. This led to the invention of the Kenzan bioprinting method.

The Kenzan method utilizes an array of stainless steel microneedles to provide a structure that the spheroids can be punctured onto to facilitate the fusion process without additional materials [22]. This method allows an actual scaffold-free tissue to be generated from various cell types for a custom-designed tissue. Regenova (Cyfuse Biomedical, Tokyo, Japan) is the only bioprinter on the tissue engineering market which utilizes the Kenzan method. Despite its young age and high cost, it has already been used to generate functional tissues from a multitude of cell types and lineages [23,24,25,26]. This device, however, places one spheroid at a time on the needles, requiring a longer printing process as the size of the tissue increases. Having the ability to build larger, more clinically relevant tissues in a shorter length of time using this method would be very beneficial for various fields of medicine and clinical research.

We hypothesize that a device which can print spheroids with a layer-by-layer method will not only reduce the manufacturing time of clinically relevant-sized tissues but also assists in advancing the field of tissue engineering by introducing an affordable alternative for printing scaffold-free, spheroid tissues. The research described here is focused on the design, construction, and testing of such a bioprinter. We also show that we were able to build a working and affordable prototype which efficiently and accurately transfers cellular spheroids to a needle array, one 3 mm × 3 mm × 1 mm layer at a time.

## 2. Materials and Methods

### 2.1. Preparation of Needle Arrays

**Figure 1:**
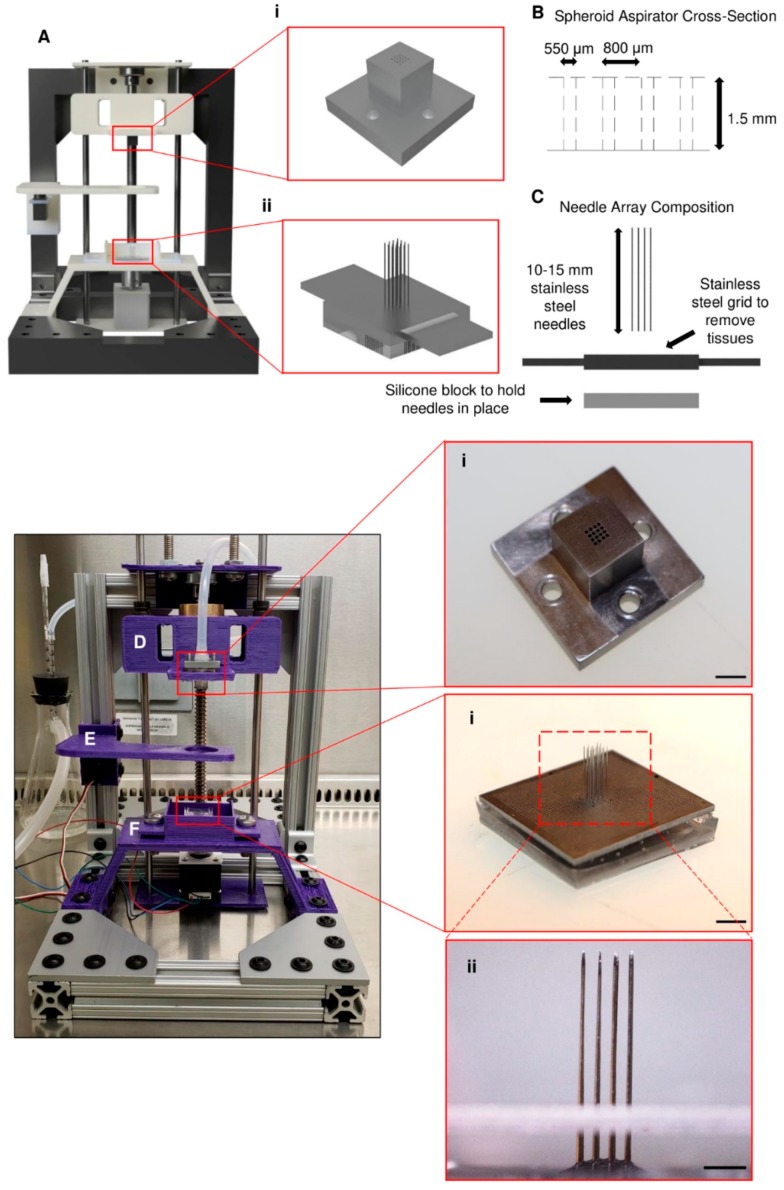
Design and construction of the bioprinter and its components. (**A**) A model of the bioprinter was first made using the CAD modeling software. The crucial components of the bioprinter are (i) the layer-by-layer print head and (ii) the needle array. (**B**) The print head has approximately 550 µm through holes cut into the bottom in a 4 × 4 arrangement, with a space of 800 µm between each hole. (**C**) The needle array is made up of 180 µm-diameter stainless steel needles placed 800 µm apart in a matching 4 × 4 arrangement. (**D**–**F**) The other components were 3D printed and are as follows: (**D**) (i) spheroid print head, (**E**) spheroid bath stage, and (**F**) needle array bath with (i,ii) needle array. Scale bars: (**D**)(i) and (**F**)(i) 5 mm; (**F**)(ii) 2 mm.

The printing method used by the utilized bioprinter relies on the accurate and precise placement of needles for the proper fusion of spheroids into a single tissue. Needle arrays (Figure 1Aii,Fi) were made using sterile stainless steel needles that were 180 μm in diameter and ~15 mm in length. To aid in the precise placement of the needles, a custom stainless steel plate (Figure 1C,Fii) that possessed an array of through holes, machined using a laser drilling technique, was used. With this plate, needles were placed into a 4 × 4 array, each with a pitch of 800 μm. Once the needles were loaded into the plate, the exposed blunt ends were placed in a silicone solution (Sylgard 184, Dow Corning, Midland, MI, USA) and cured at 70 °C for 2 h to hold the needles in place during the printing process effectively. After curing, the needle array assembly was autoclaved and then kept until ready for use with the bioprinter. 

### 2.2. Bioprinter Design and Construction

The bioprinter (Figure 1A) was designed to fit easily into a biosafety cabinet and operate under sterile conditions. It is 10 inches tall and 8 inches wide, with a depth of 13 inches. The bioprinter was entirely modeled in Fusion 360 (Autodesk) before manufacturing the required pieces for the device. The model of the bioprinter as well as the built bioprinter are shown in Figure 1. The bioprinter consists of three main parts: a spheroid print head (Figure 1D), a spheroid bath stage (Figure 1E), and a needle array bath (Figure 1F). The spheroid print head (Figure 1Ai,Di) was designed to hold a 4 × 4 array of spheroids in a single layer. Each spheroid is approximately 800 µm apart when held on the end of the print head. The print head was machined from 316 stainless steel, and through holes were cut into the bottom of the print head with a diameter of approximately 550 µm, allowing the needles to pass through but preventing spheroid aspiration (Figure 1B). The beads were picked up by a vacuum pump (12V vacuum pump, SparkFun, Niwot, CO, USA) connected to the print head. The spheroid bath stage functioned to hold the reservoir/container of beads during the printing process. The reservoir consisted of a watch glass made from polytetrafluoroethylene (PTFE), whose concave bottom allowed for the beads to collect in the center after each layer was picked up. The beads were printed onto the needles which were held inside the needle array bath. Each piece of the printer can either be sterilized by autoclave or ethylene oxide.

### 2.3. Bioprinting Process

**Figure 2:**
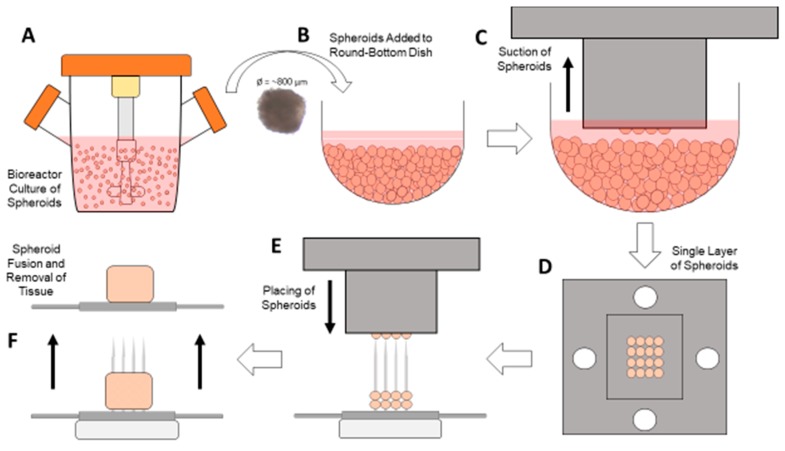
Bioprinting process. Spheroid culture can take place in (**A**) a bioreactor or a standard round-bottom culture plate, and once the spheroids are approximately 800 µm in diameter, they can be placed in (**B**) the round-bottom dish on the spheroid stage. Once the desired number of spheroids are placed in the dish, (**C**) the print head is lowered down to the center of the dish, and the vacuum is turned on, capturing (**D**) a single layer of spheroids. With the vacuum still on, the print head is lowered down to the needles (**E**), placing the spheroids on them. After the required number of layers is achieved, (**F**) the spheroids are allowed to fuse and then later removed for further processing.

This scaffold-free bioprinter was controlled using an Arduino (Arduino Uno Rev3, Arduino, Somerville, MA, USA) and a custom-written script. It controlled the stepper motor (NEMA 14, Lin Engineering, Morgan Hill, CA, USA) for moving the print head, the servomotor (5V servomotor, Arduino, Somerville, MA, USA) for moving the spheroid stage, the vacuum pump for aspirating the spheroids, and the end stop (Mechanical End Stop, SparkFun, Niwot, CO, USA) mechanism for calibrating the stepper motor prior to beginning the printing process. The bioprinter was designed to first automatically calibrate to ensure that the print head is in the proper position before beginning. Once it has calibrated, the Arduino program prompts the user to load the spheroids into the spheroid stage in the round bottom plate (Figure 2B). Next, the user is asked to input the exact number of layers to print, between one and five. Once this value is entered, the printing process begins (Video S1). First, the print head is lowered down into the spheroid container, and the vacuum pump is activated to aspirate one layer of spheroids (Figure 2C,D). While the vacuum is still on, the print head is raised out of the container, and the spheroid stage is moved out of the way. The print head with spheroids is lowered straight down onto the needles in the array bath (Figure 2E). Once the print head is placed in the correct position, the vacuum pump is turned off, and the spheroids are left on the needles. This process is repeated until all layers have been placed on the needles, with each subsequent layer being placed ~800 μm higher than the previous layer. After all layers are completed, the array bath is removed from the system and placed in the incubator for at least 24 h for fusion to take place. Once the spheroids have adequately fused, the stainless steel plate is used to assist in the removal of the fused tissue for further processing (Figure 2F).

### 2.4. Vacuum and Print Head Testing

Glass beads were used to determine the efficiency of the vacuum and print head system. Glass beads approximately 700 µm in diameter (±10% diameter, Zirconia Beads, BioSpec Products, Bartlesville, OK, USA) were used in place of cultured spheroids because of the number of tests that needed to be completed and the non-sterile conditions of the testing area. This diameter was chosen because it is similar to the 800 µm diameter of the spheroids that our lab has previously produced [27]. These beads were held on the spheroid stage in the PTFE container. To help counteract aggregation during the testing process, the beads were placed in a solution of phosphate-buffered saline with 0.1% Tween 20 (PBST), which also allowed to simulate spheroids in media. To determine the different efficiencies of these experiments, the print head was lowered onto the spheroid stage and the vacuum was turned on, which allowed the print head to pick up the beads. After a given time, the number of aspirated beads were counted and calculated as a percentage of the total number of holes available to collect the beads (e.g., 15 beads aspirated into an array of 16 possible holes equates to an efficiency of 93.8%).

### 2.5. Preparation of Alginate Beads

Alginate beads were chosen as a reasonable spheroid surrogate to test the ability of the bioprinter to place spheroids on the needle array effectively. As mentioned previously, spheroids that would be used with this system are approximately 800 µm in diameter, so the alginate beads needed to have a similar diameter. These beads were generated by adding a solution of 1.5% sodium alginate (Cat. No. 218295, MP Biomedicals, Irvine, CA, USA) in deionized water (DI) water to a 100 mM solution of calcium chloride (Cat. No. 4901, Sigma Aldrich, St. Louis, MO, USA) in DI water [28]. The alginate solution was added using a 22-gauge serological needle dropwise into the calcium chloride solution which was being stirred using a magnetic stir bar. The beads were separated according to their approximate diameter and measured using image analysis.

### 2.6. Preparation of Human Induced Pluripotent Stem Cell (hiPSC) Spheroids

Human induced pluripotent stem cells (hiPSCs) were cultured on Matrigel-coated dishes at 37 °C under 5% CO_2_ with mTeSR (Stem Cell Technologies, Vancouver, BC, Canada). Fresh medium was added daily until the cells reached 90% confluency. The cells were seeded and grown in suspension according to the manufacturer’s protocol [29]. Briefly, the cells were passaged by treating with Gentle Cell Dissociation Reagent (Stem Cell Technologies, Vancouver, BC, Canada) for 7 min. The cells were carefully scraped off the dish and seeded at a density of 5 × 10^5^ cells per mL in 30 mL of mTeSR 3D Seed Media (Stem Cell Technologies, Vancouver, BC, Canada). This solution was then cultured in a 125 mL shaker flask at 70 rpm with daily media additions of 3.4 mL. Prior to their use with the bioprinter, the spheroids were filtered through a 500 µm reversible strainer (pluriSelect, El Cajon, CA, USA) and collected in 5 mL of mTeSR.

### 2.7. Statistical and Image Analysis

Data are shown in the form mean ± standard error of the mean (SEM). Significance was chosen as p < 0.05. This was determined using both one- and two-way analysis of variance (ANOVA) along with the Student’s t-test. These analyses were performed utilizing Microsoft Excel’s data analysis software package.

For image analysis, ImageJ (Version 1.52o with the addition of plug-ins for colocalization (Colocalization Finder, Version 1.2, Institut de Biologie Moleculaire des Plantes, Strasbourg, France) was used to estimate the overlap coefficient between each layer of beads and to generate representative pictures. Also, it was used in the diameter measurements of the alginate beads that were synthesized. Images and videos in this experiment were taken with a stereomicroscope (Olympus SZ61, Olympus, Center Valley, PA, USA), a phase-contrast microscope (AMG EVOS FL Imaging System, Thermo Fisher Scientific, Waltham, MA, USA), and a Canon camera (Canon EOS Rebel T7i, Canon, Tokyo, Japan).

## 3. Results 

### 3.1. System Testing for Efficiency of Sphere Capture and Transfer

**Figure 3:**
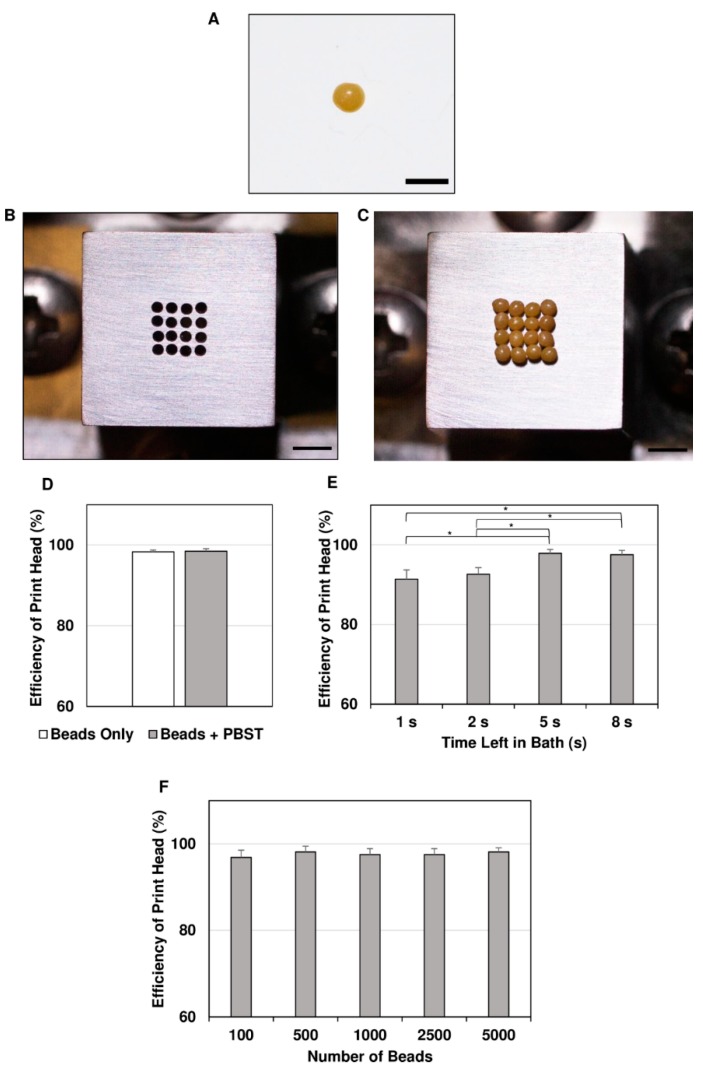
Vacuum and print head capture efficiencies. A substitute was chosen for spheroids because of the non-sterile conditions and multiple tests that needed to be performed. (**A**) Zirconia beads with a diameter (700 ± 70 µm) equivalent to that of the spheroids were used. (**B,C**) The print head (B, seen from bottom) was used to pick up (C) a 4 × 4 array of beads in a dry setup. After the print head was placed in the desired container of beads, the vacuum was turned on for 10 s. At the end, the number of beads captured was counted, and (**D**) the efficiencies were calculated. (**E**) Then, the optimal time for the vacuum to be on at which most beads would be captured was determined to be 5 s (* p < 0.05, n = 13 for each); (**F**) how the number of beads in the container affected the precision of the bioprinter was also determined. Scale bars: (**A**) 1 mm, (**B**), and (**C**) 2 mm.

To test the ability of the vacuum pump system and print head to pick up spheroids, glass beads similar in diameter to the average spheroids that would be used with this printing setup were used. These beads, according to the supplier’s specifications, had a diameter of 700 ± 70 µm on average (Figure 3A), which is very similar to the ~800 µm diameter of the spheroids that would be used. The efficiency of the system was determined by using two separate containers (Beads Only and Beads + PBST) of spheres which were picked up by turning on the vacuum pump system for 10 s at a time, removing the print head (Figure 3B) from the container, and then counting how many spheres had been picked up. After collecting the beads (Figure 3C) various times (n = 28 for Beads Only; n = 18 for Beads + PBST), the efficiencies of sphere capture were determined to be 98.3 ± 0.5% for Beads Only and 98.4 ± 0.6% for Beads + PBST (Figure 3D). 

To further optimize the printing process, the time during which the print head remained in the bead container was varied (1 s, 2 s, 5 s, and 8 s) to determine the optimal time needed to pick up most of the beads. For each time, the corresponding efficiencies (n = 13 for each time point) were calculated (Figure 3E). From this test, it was determined that 5 s was the shortest and most efficient time, with an efficiency of 97.8 ± 0.9% which was significantly different (p < 0.05) from those measured for times of 1 s (91.4 ± 2.3%) and 2 s (92.6 ± 1.7%) but did not change significantly when the time increased to 8 s (97.5 ± 1.1%) or 10 s. 

In addition, due to the fact that as spheroids are removed, more empty space in the spheroid container would be present, a test was done to determine if the number of beads within the container affected the capture efficiency. Beads were added to the dish at amounts of 100, 500, 1000, 2500, and 5000, estimated on a mass basis. The print head was used to capture beads a number of times (n = 10 for each group), and the subsequent efficiencies were determined (average efficiency: 97.6 ± 0.2%) (Figure 3F). There was no significant difference between any amounts of beads tested, showing that as the number of beads decreases in the container, the efficiency does not change.

**Figure 4:**
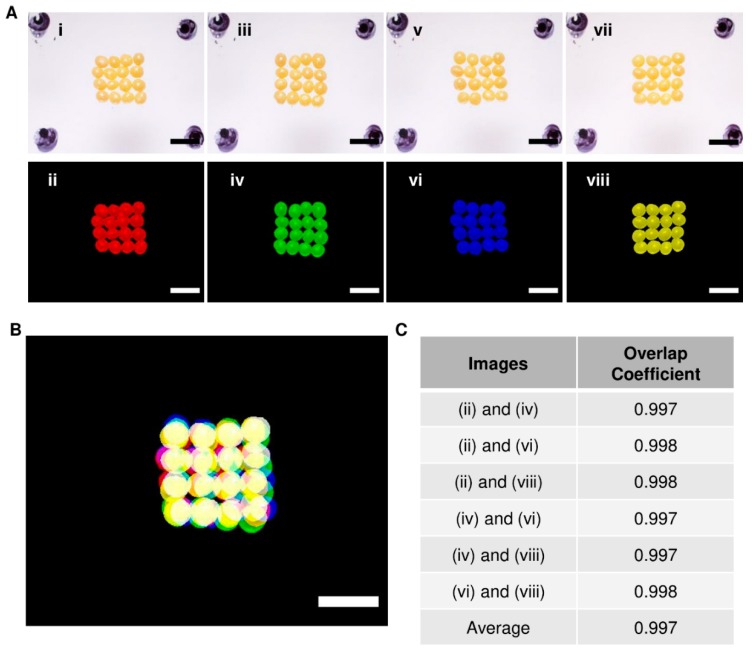
Bead layer-by-layer overlap coefficient determination. To determine the precision of the bioprinter, (**A**) layers of beads were added to a predefined area (upper panels), and each run was assigned a color (lower panels). (**B**) Images from individual runs were overlapped using ImageJ. The yellow color indicates the overlap of the beads. (**C**) The overlap coefficient was determined (Colocalization Finder for ImageJ) using the images, and the average overlap coefficient was found to be 0.997. Scale bars: 2 mm.

The repeatability of the bioprinter ability to place the beads in the same area with each subsequent layer was tested to ensure that the spheroids could accurately be placed on the needles when generating tissues. This was determined by placing a full layer of beads into a pre-determined area on a layer of silicone multiple times. After every single layer was placed, an image was taken of the beads, with each placed layer given a different color (Figure 4A). A composite image of each layer aligned together was generated to demonstrate that the bioprinter could place the spheroids in the same area, made apparent by the overlap in colors (see yellow color) (Figure 4B). To estimate the degree of overlap between the layers, a colocalization tool in conjunction with ImageJ was used to calculate the overlap coefficient for each combination of any two layers (Figure 4C). The overlap coefficients between any two layers were greater than 0.99, showing that each layer closely overlapped with the others.

### 3.2. Alginate Bead Formation and Print Head Testing

**Figure 5:**
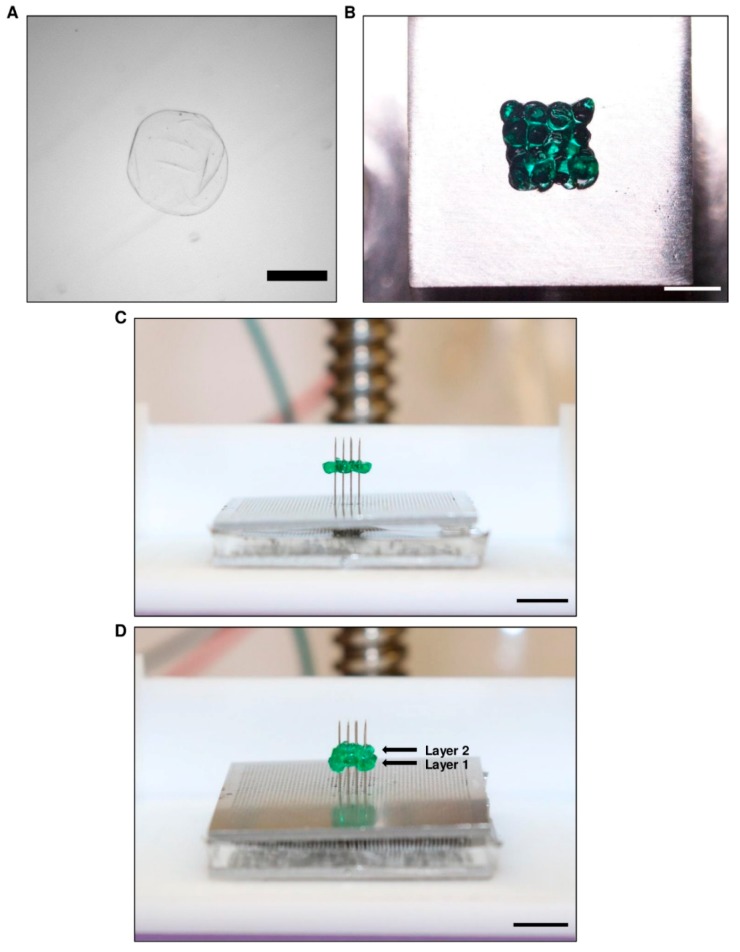
Bioprinter testing with alginate beads. Soft alginate beads were used as a spheroid surrogate as they could easily be punctured and placed on the bioprinter needles. (**A**) The beads were synthesized at an average diameter of 851 ± 15 µm, which was similar to the spheroid diameter. (**B**–**D**) Picture (**B**) showing beads aspirated onto the end of a 4 × 4 arrangement print head and (**C**) printed onto the needles in a single layer. (**D**) Two layers were also tested to ensure that multiple layers could be printed. Scale bars: (**A**) 500 µm; (B) 2 mm; (**C**) and (**D**) 5 mm.

Small beads made from alginate were chosen as a surrogate for cellular spheroids for testing the bioprinter’s ability to print onto the needle arrays correctly. Alginate beads were synthesized using a dropwise technique (Video S2) and separated according to their diameter. Once these beads were separated, they were collected into a single dish and measured using phase-contrast microscopy (Figure 5A). The average diameter of the beads (n = 27) was determined to be 851 ± 18 µm, which is similar to the desired diameter of 800 µm. The beads were then picked up with the print head (Figure 5B) and transferred to the needles (Figure 5C) using the bioprinting process (Video S3). Placing a single layer of beads took approximately 45 s. 

Additionally, to determine how effectively the bioprinter could print multiple layers of spheroids, two layers were printed onto the needles (Figure 5D; Video S4). From these data, it was seen that the bioprinter could successfully print the alginate beads in multiple layers. Our results clearly show that multilayer printing of spheroids is possible using our newly designed bioprinter.

### 3.3. Proof-Of-Concept Testing with hiPSC Spheroids

**Figure 6:**
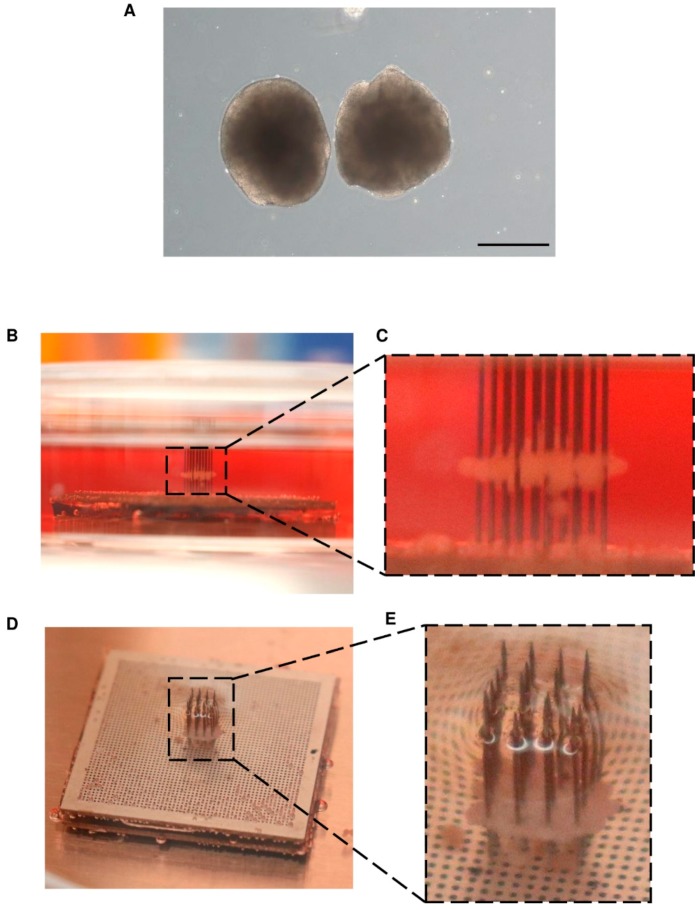
Proof-of-concept testing with human induced pluripotent stem cells (hiPSC) spheroids. hiPSC spheroids were cultured using a rotating culture flask method. (**A**) The spheroids were grown to an average diameter of 718 ± 77 µm. Once enough spheroids were available to be used with the bioprinter to create a single layer, they were loaded into the bioprinter and placed onto the needles. (**B**,**C**) Side view of the hiPSC spheroid layer after placement onto the needles. (**D**,**E**) Top view of the hiPSC spheroid layer. The ability to print cellular spheroids onto the needle array confirms the efficiency of the layer-by-layer printing technique presented here.

We confirmed the potential of our bioprinter to place cellular spheroids onto the needles in a layer-by-layer fashion. For this, hiPSC spheroids were grown to a similar diameter of, on average, 718 ± 77 µm (Figure 6A, Image S1) and added to the round-bottom container. The bioprinter was started, and a single layer of spheroids was picked up and placed onto the needles. The top (Figure 6B,C) and side (Figure 6D,E) view images show a single layer of spheroids on the needles. These data clearly show that the bioprinter was effectively able to aspirate and transfer a single layer of cellular spheroids onto the needles.

## 4. Discussion

In this study, we were able to construct a novel and affordable bioprinting device with the ability to produce scaffold-free tissues through the utilization of layer-by-layer printing of cellular spheroids. This device could allow the enhanced production of viable tissues to be used in drug testing, translational research, and various clinics. 

With the design and development of the layer-by-layer print head, efficiently picking up the spheroids from a reservoir was paramount for the overall success of the bioprinter. Through testing with beads of size similar to that of the spheroids, we were able to achieve nearly 98% efficiency when picking up the beads in a single 4 × 4 layer. Although the print head designed in this project could only transfer a maximum of 16 beads or spheroids, changes can easily be made to the number and arrangement of holes in the bottom of the print head. This would allow for the construction of both larger tissues as well as custom arrangements to be used in different applications where 3D models of the native tissue microenvironment are needed [30,31]

In addition, we were able to show the reliability of the bioprinter to print in a localized position repeatedly. The lowest overlap coefficient calculated between any two layers was 0.997. The closer to 1.0 this value is, the more colocalized the beads of different layers are. This corresponded to the precise placement of the spheroids on the needles when testing with hiPSC spheroids. This precision will allow for the proper alignment of layers in the construction of native tissue microenvironments as well as any cellular gradients found within tissues. The reproducibility and speed of printing viable tissues are critical for the success of bioprinting in the clinical environment [32,33]. The only commercially available spheroid printing device, Regenova by Cyfuse Biomedical, was used to generate a tubular, vessel-like structure that was made from approximately 500 spheroids [26]. Itoh et al. noted that to place so many spheroids onto the needles, it took the bioprinter approximately 1.3 h (78 min). This corresponds to approximately six spheroids printed per minute. The bioprinter we present here can print 16 spheroids per minute, that is, an over 250% increase in the printing rate of spheroids. Besides, given the success and efficiency of the print head in picking up and transferring a full layer of spheroids to the printing surface, we can quickly increase the number of holes in the bottom of the print head to accommodate more spheroids and thus produce larger tissues.

Compared to other methods which utilize single-spheroid printing, a process requiring a multitude of precise positioning controls and visual analysis, this layer-by-layer method allows to straightforwardly pick up and place the spheroids [22]. This not only leads to a faster printing speed but also decreases the overall cost of our device, which totaled approximately $2000, a significant reduction in price compared to the only commercially available option.

Despite the advancements in scaffold-free spheroid printing that have been shown here, there remain a few limitations in this presented design. One in particular is the inability to customize the size and shape of the tissues. Future iterations will need to have the ability to print different tissue shapes (e.g., vessel, round, etc.) to expand the capabilities to other fields of tissue engineering. Also, the current design is limited to holding only one type of spheroid for printing. Having the choice to print layers of different types of spheroids would increase the customization potential. Further design changes could allow for the placement of multiple spheroid reservoirs on the spheroid bath stage of the bioprinter. 

## 5. Conclusions

In conclusion, we present here a custom device for printing spheroids using a novel layer-by-layer method. This bioprinter can efficiently pick up and transfer spheroids to the printing stage in a single, complete layer, decreasing the printing time of large tissue constructs. With this precise and accurate transfer of spheroid-like beads to the printing surface, custom tissues can easily be constructed on the needles in future experiments. Also, we were able to prove, using hiPSC spheroids, that we can successfully print a single layer of spheroids onto the needle array printing surface. In addition, we were able to build and manufacture this device at an affordable price, making it easier for other researchers to use the technology if brought to the market. Although there are additional design considerations that can be incorporated into future iterations, this device helps to advance and improve the growing area of scaffold-free bioprinting and could one day become a go-to method in tissue engineering.

## Supplementary Materials

The following are available online at https://www.mdpi.com/2072-666X/10/9/570/s1, Video S1: Layer-by-layer bioprinting process. Video S2: Alginate bead synthesis. Video S3: Printing of alginate beads onto the needle array. Video S4: Printing of two layers of alginate beads onto the needle array. Spreadsheet S1: Major data for figures including efficiency data, colocalization data, and alginate bead diameter data. Image S1: Immunolabeling of hiPSC spheroids.
