# Supplementary figures and images for: Scaffold-Free Bioprinter Utilizing Layer-By-Layer Printing of Cellular Spheroids
Source: Micromachines (Basel). 2019 Aug 29;10(9):570. doi: 10.3390/mi10090570 (PMC6780220; doi:10.3390/mi10090570)

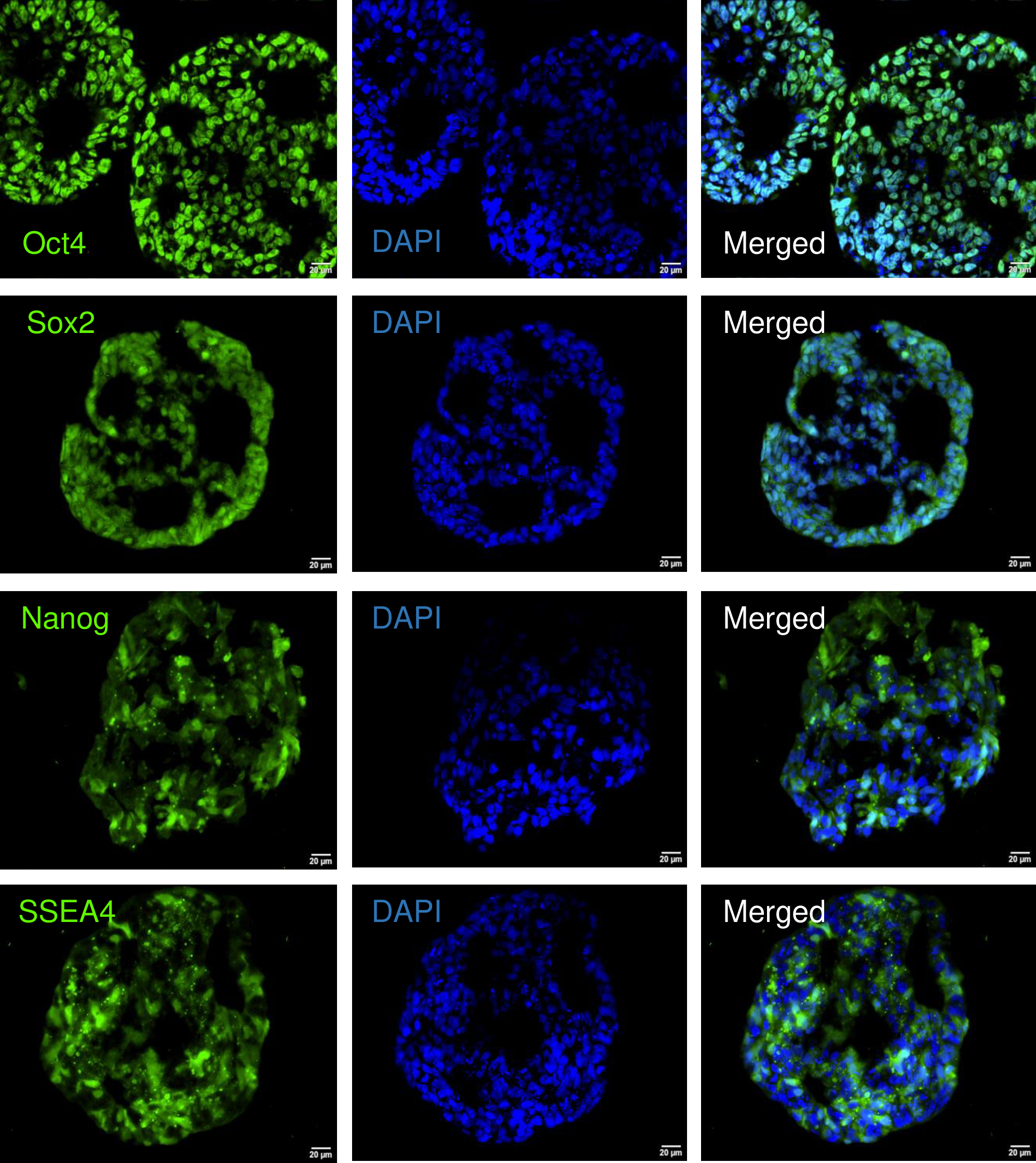

Supplement: Supplementary file 1 [file micromachines-10-00570-s001.zip › Images S1.tif]
